# Synthetic Aperture Radar Imaging for Burn Wounds Diagnostics

**DOI:** 10.3390/s20030847

**Published:** 2020-02-05

**Authors:** Amani Yousef Owda, Majdi Owda, Nacer-Ddine Rezgui

**Affiliations:** 1 Department of Electrical and Electronic Engineering, University of Manchester, Sackville Street Building, Manchester M13 9PL, UK; 2 Department of Computing and Mathematics, Manchester Metropolitan University, Chester Street, Manchester M1 5GD, UK; m.owda@mmu.ac.uk; 3 Department of Engineering, Manchester Metropolitan University, Chester Street, Manchester M1 5GD, UK; nrezgui@hotmail.com

**Keywords:** dressing materials, synthetic aperture radar, burn wounds, microwave imaging, reflectance

## Abstract

The need for technologies to monitor the wound healing under dressing materials has led us to investigate the feasibility of using microwave and millimetre wave radiations due to their sensitivity to water, non- ionising nature, and transparency to dressing materials and clothing. This paper presents synthetic aperture radar (SAR) images obtained from an active microwave and millimetre wave scanner operating over the band 15–40 GHz. Experimental images obtained from porcine skin samples with the presence of dressing materials and after the application of localised heat treatments reveal that SAR images can be used for diagnosing burns and for potentially monitoring the healing under dressing materials. The experimental images were extracted separately from the amplitude and phase measurements of the input reflection coefficient (S_11_). The acquired images indicate that skin and burns can be detected and observed through dressing materials as well as features of the skin such as edges, irregularities, bends, burns, and variation in the reflectance of the skin. These unique findings enable a microwave and millimetre-wave scanner to be used for evaluating the wound healing progress under dressing materials without their often-painful removal: a capability that will reduce the cost of healthcare, distress caused by long waiting hours, and the healthcare interventional time.

## 1. Introduction

**Figure 1:**
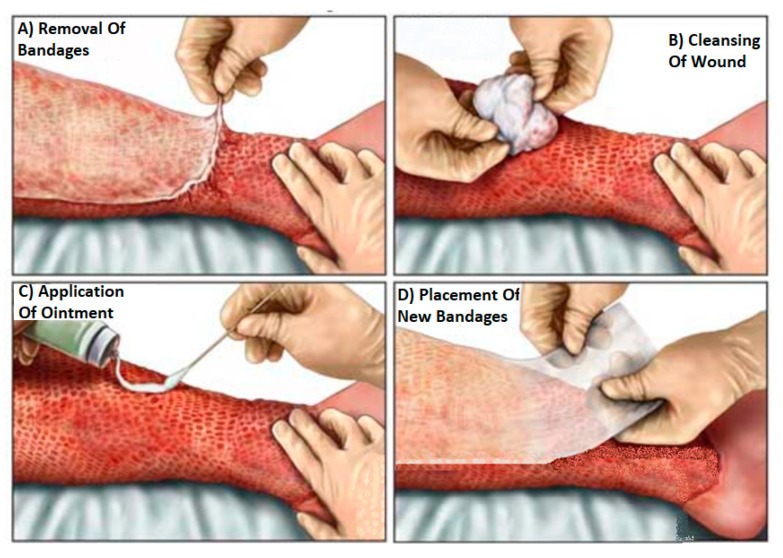
Visual inspection for assessing burn wounds [28]. This involves the removal of dressing materials as illustrated in (**A**), cleaning the wounds as in (**B**), applying medicinal cream as in (**C**), and placement of new dressing materials as in (**D**).

Burns are a very common cause of injury with over 3750 people under 15 years requiring treatment a year in the UK and costing the National Health Service (NHS) millions of pounds [1]. Visual inspection is the current medical practice for assessing burn wounds [2,3]. This practice involves the removal of dressing materials for monitoring the wound healing progress and detecting the signs of infection as illustrated in Figure 1. The relative demerits for this practice are being uncomfortable and painful to the patient (especially for children) [2]. As an alternative to the current medical practice, technologies are emerging to enhance the medical assessment of burn wounds such as terahertz imaging [4,5,6,7], optical coherence tomography [8,9,10,11,12], ultrasound imaging [13,14,15,16], infrared imaging [17,18,19,20], and microwave and millimetre wave (MMW) sensing [2,21,22,23,24,25,26,27]. The latter is the subject of the research in this paper.

The dependence of reflection and emission of terahertz radiation (THz > 300 GHz) to variation in the water content in biological tissue makes terahertz imaging feasible for non-invasive diagnosis of burn wounds [4,5,6,7]. The terahertz time-domain spectroscopy (THz-TDS) introduced by Arbab et al. [6] was used to obtain in vivo images from second and third degree burn wounds from rats. This study indicates a possible capability for THz-TDS to differentiate between partial thickness and full thickness burn wounds. The in vivo reflectivity measurements conducted by Arbab et al. [7] indicate that the reflectivity of the burned skin is higher than that of the unburned skin. This can be supported by other in vivo terahertz images obtained from the rats’ skin by Tewari et al. [4]. This study shows dynamic variations in skin reflectivity following the burning process, an increase in the skin reflectivity was observed in the burned skin as a result of increased the water concentration due to the post-injury inflammatory response, whereas, lower reflectivity was observed in the surrounding unburned tissue. Although, terahertz imaging seems to be a promising technology for burn wound [5], the low penetration depth in the human body of THz radiation is the main limitations. Due to the limited penetration capability of THz radiation, magnetic resonance imaging (MRI) has been used with THz imaging to assess the burn wound as it can provide comprehensive information about the tissue water content (TWC) and the dynamic behaviour of the water in burn wound over a depth greater than 258 μm [29,30]. The work conducted by Lohmann et al. [31] indicates that the spatial resolution of the MRI can be enhanced using magnetic field strength of 7.0 Tesla and various imaging protocols. Although MRI can provide useful information to assess the severity of burns, there are limited measurements conducted in this area and this might be due to the very high cost of this technology. Therefore, it is still early to assess how much MRI might be useful for assessing the severity of the burn wound.

Optical coherence tomography (OCT) is a high-resolution (~10 µm) in vivo surface imaging that is suggested for use in medical applications and in the non-invasive diagnosis of burn depth [8,9,10,11,12]. The in vivo study conducted on female rats by Srinivas et al. [9] suggests that polarization-sensitive optical coherence tomography (PS-OCT) might be feasible to assess the burn wound depth in the first two days only as increasing the water content and exudates due to edama formation present the main limitation. The high-resolution cross-sectional images obtained from the high-speed fiber-based PS-OCT by Park et al. [10] indicate that the detectable amount of collagen content in the burn surface might be useful to assess the degree of the burn as severe burns have higher collagen content compared with superficial burns. The in vivo study applied on two human patients having superficial and full thickness burn wounds by Kim et al. [11] indicates that the three-dimensional images obtained from PS-OCT can characterise the burn wound based on vasculature and birefringence. Although, the in vivo study applied in a mouse model conducted by Zhao et al. [8] indicates that spectroscopic optical coherence tomography (SOCT) shows significant differences in the spectra associated with the depth of the burn. However, this approach is not sufficient to assess the deep burns, as the penetration capability is only a few hundred microns from the skin surface [8]. According to Rayleigh [12,32], scattering is inversely proportional to the wavelength and this makes the phase reconstruction of an image at depth, through the overlying tissue difficult.

Ultrasound waves are high-frequency sound waves above the human hearing (typically greater than 20 KHz). These waves are used to generate images of the internal tissue and organs of the human body by using the reflected pulse echoes. The basic elements of the traditional ultrasound scanner are; a transducer (a hand-held probe) that is located and moved manually to scan the target area of the human body, and a water-based gel that is used to enhance the coupling between the probe and the human body [33]. Ultrasound imaging has been used in the assessment of burn wounds [13,14,15,16,34]. The study conducted by Rippon et al. [13] indicates that ultrasound imaging at 20 MHz can visualise porcine skin (in vivo) and human tissue (cadaver) to a depth of 25.0 mm. The in vivo study conducted on six Yorkshire pigs by Gnyawali et al. [14] indicates that ultrasound imaging combined with a laser speckle might be an effective tool for the non-invasive diagnosis of burn wounds. In addition, ultrasound imaging seems to be an efficient tool for providing qualitative information about the burn depth [15] and the collagen content [13]. Although non-contact ultrasound imaging has been developed to assess the burn depth [16], it has not been adapted clinically [34]. The main limitations of the ultrasound imaging are artefacts and clutter generated from unwanted reflected echoes [14].

Over the infrared frequency band, a thermogram analyser (infrared camera) was used to examine the healing progress of the burn wound by estimating the surface area of the burn based on temperature detection [18]. In vivo thermographic images obtained from 65 patients having burns injury by Liddington and Shakespeare [17] indicate that thermography might be used to assess the burn depth within 72 h post injury, as deep burns expressed a significant variation in the temperature after 48 h to 72 h after the burn injury. The results presented in this study are in good agreement with the results obtained from another study conducted on two female pigs by Miccio et al. [19]. Furthermore, the study conducted by Ruminski et al. [20] introduces the active dynamic infrared thermal imaging as a technique to assess the burn depth. Although infrared imaging has been introduced as a non-invasive technique for assessing burn wound [17,18,19,20], it has not been adapted clinically due to the following limitations: (1) Infrared imaging relies on variation in thermodynamics temperature and this might be measured an error due to the presence of exudates and blood flow. (2) Infrared radiation has a very low penetration capability. Therefore, it provides information about the surface area of the skin only.

Microwave and MMW radiations are very sensitive to the variations in water content in biological tissues. Since burn wounds cause significant variations in the water content of the tissue, as an immediate response to burn injury (that produces blood and lymph fluid), the radiation is useful for assessing bandaged wounds [24,35]. In the literature survey, different technologies are suggested for assessing burn wounds such as terahertz imaging [4,5,6,7], optical coherence tomography [8,9,10,11,12], ultrasound imaging [13,14,15,16], and infrared imaging [17,18,19,20]. Although the results obtained from these technologies are promising in terms of assessing burn wounds depth, none of these technologies successfully assesses the wound healing progress without the removal of dressing materials. Currently, there are no methods for effectively assessing the wound healing progress without removing the dressing materials. A technique that could identify the healing state of a burn wound under dressing materials is of great interest to patients, healthcare professionals, the National Health Service, and the private healthcare industry. It would reduce the pain, anxiety and distress caused by wound dressing changes, as well as reducing healthcare interventional time [2]. Because electromagnetic radiation at microwave and millimetre wave frequencies can propagate through typical dressing materials with little attenuation (less than 0.85 dB for both Ka and W bands [2]), these bands of the electromagnetic spectrum are promising for assessing bandaged wounds. 

Microwave and MMW radiations are both non-ionising radiations that are capable of providing highly localised measurements. Knowledge of this and the transparency of bandages in the MMW region has led Essen et al. [24] to scan a phase image of the healing of the scar using in-contact active MMW scanner at 94 GHz. Meanwhile, Gao and Zoughi [25,26] conducted reflectivity measurements on pigskin samples. These measurements [25,26] suggested that MMW reflectometry could be used as a non-invasive in-contact technique to distinguish between unburned and burned skin having different degrees of burn injuries, and more importantly [26] indicates the potential of using SAR images to detect burn wound under dressing materials over the band (50–75) GHz. Although, the results obtained from this study [26] are promising and illustrate the potential of using active MMW radiation for detecting burns under dressing materials. These results [26] were unable to show features of the skin and dimensions of the burn under dressing materials.

In our previous research we have constructed the half space electromagnetic model (a simulation model) [22] to assess the feasibility of using radiometry (passive sensing technology) for non-invasive diagnosis of diseased skin. Simulations results from the half space electromagnetic model in [22] show that the emissivity of the skin varies with the skin water content and this could be used as a metric to detect and monitor malignancy, eczema, psoriasis and burn wounds. In [23,36,37,38] we have measured the emissivity of the human skin on different groups of healthy participants over the frequency band (80–100) GHz. Radiometric measurements in [36,37] indicate that there is a clear signature for the human skin over the millimetre-wave band and this signature can be detected using radiometry. Radiometric measurements in [23] show a well define contrast in the emissivity of the human skin between thinner and thicker skin regions for both genders. Further, emissivity measurements in [38] indicate that radiometric sensitivity is sufficient to distinguish between normal skin and skin after the application of gel. These measurements [23,36,37,38] reveal that variations in the human skin emissivity are related to the skin thickness and water content. As burn wounds affect significantly the water content of the skin and the skin thickness; we have investigated the feasibility of using a single channel radiometry (passive sensing) to distinguish between unburned and burned skin in [2,27]. Radiometric measurements obtained from a chicken phantom using a 95 GHz radiometer in [2] indicate that there are well define differences in the mean emissivity values between unburned and burned skin and these differences are observable through dressing materials, indicating the feasibility of using radiometry to detect changes in tissue emissivity under dressing materials. Further, Radiometric measurements in [27] indicate that millimeter-wave radiometry generates a clear signature of porcine skin burns and this could be used as a non-contact method to determine the severity of the burns and the degree of the burns. As an active sensing technology has the advantage of higher penetration capability compared with the passive sensing technology; a mono-static radar system in [21] was used for measuring the propagation path length and the thickness of the dressing materials at different states (dry, wet, and with medicinal cream). Experimental measurements in [21] indicate that the mono-static radar system is capable to provide precise information about the thickness of the dressing materials using the distance between two reflection peaks. In this paper an active microwave and millimetre-wave scanner (active imaging technology) combined with SAR image processing algorithm was used to scan images from porcine skin samples over the frequency band 15–40 GHz unlike other studies [2,21,22,23,27,36,37,38] that focus on measuring the mean emissivity values of the skin and the thickness of the dressing materials. The experimental images presented in this paper were extracted from the phase and the amplitude measurements of the input reflection coefficient. The key innovation in this work is in recognizing features of the skin under dressing materials i.e., (burn width, skin reflectance, irregularities in the skin surface, edges and bends on the skin, and shape of burn) as well as assessing the feasibility of the potential of using active sensing technology for evaluating the wound healing progress under dressing materials.

This paper presents experimental images obtained from porcine skin samples with and without the presence of dressing materials and before and after the application of localised heat treatments. Experimental images over the band 15–40 GHz in this paper are aiming to show burns and features of the skin under dressing materials to assess the feasibility of using microwave and MMW radiations to monitor bandaged burn wounds. The images presented herein were extracted from both the amplitude and the phase measurements of the input reflection coefficient (S_11_) unlike other studies [24,26] that were based on either the phase or the amplitude measurements of the complex scattering parameters. To the best of the authors’ knowledge, this study represents the first demonstration of skin features under dressing materials, i.e., (burn width, skin reflectance, irregularities in the skin surface, edges and bends on the skin, and shape of burn). The study also provides a comparison for the first time between phase images and amplitude images and proves that useful information can be extracted from the two measured physical quantities, i.e., (amplitude and phase).

The remainder of the paper is structured as follows. Section 2 describes the experimental methodology applied on porcine skin samples and the methodology of data processing, Section 3 presents the experimental results obtained from porcine skin samples, Section 4 discusses the experimental images and highlights motivations for future directions, and finally Section 5 draws the overall conclusions.

## 2. Materials and Methods

### 2.1. Porcine Skin Samples

Porcine skin samples were used for scanning images of the skin with and without the presence of dressing materials and before and after the application of localised heat treatments. A motivation for measuring porcine skin in this research is that it has structural and functional similarities to the human skin [39,40]. Porcine skin samples used in this research were purchased from an abattoir. The samples were taken from pigs having ages ranging from six to eight months and average weights from 60 kg to 70 kg. The samples were taken from the back region of healthy animals. This region was chosen since it is free from hair follicle and sweat glands. The study was approved by the ethics committee of Manchester Metropolitan University under ethics reference no: SE1617114C.

### 2.2. Selection of Frequency Band

The microwave and MMW scanner presented in this research is effective over the band (15–40) GHz. This band provides wavelengths in the range of 7.5 mm to 20 mm and a range resolution of 6 mm or less based on the medium. The penetration depth of the radiation over this band is ranging between 0.7 mm (at 40 GHz) to 2 mm (at 15 GHz) [41]. These characteristics make the active scanner a good candidate to penetrate dressing materials and detect features of the skin under dressings as well as scanning plane surfaces such as the skin.

### 2.3. Experimental Setup

**Figure 2:**
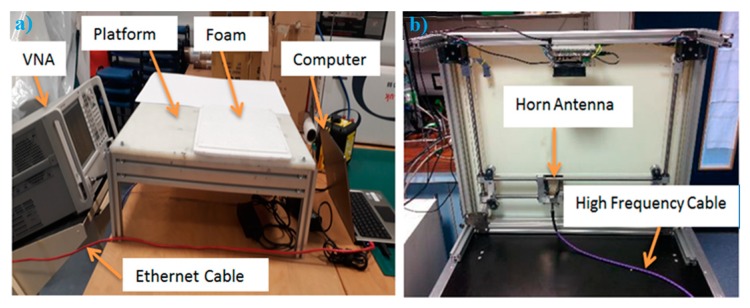
The front view of the active microwave scanner (**a**) and the bottom view of the scanner (**b**) The scanner has 6 mm theoretical range resolution and 25 GHz bandwidth.

A two-dimensional imaging scanner combined with a Matlab programme was implemented and developed by Rezgui et al. [42,43,44]. The scanner was effective over the band (15–40) GHz and it consisted of a two port vector network analyser (type: N5227A, manufacture: Keysight Technologies). The vector network analyser (VNA) was used to illuminate the sample under test (in this research porcine skin) with coherent microwave radiation having a transmission power level less than 1 mWatt. This level was chosen as it is within the safety limits and the recommendations of the Institute of Electrical and Electronics Engineers (IEEE) [45]. The VNA was connected through a high-frequency cable to a standard gain pyramid horn antenna (type: WR-34 standard waveguide, manufacturer: Flann Microwave) effective over the band 15–40 GHz (Ku, K, and Ka). The antenna was used for transmission and reception purposes (transceiver). The porcine skin sample was placed and aligned on a platform to be scanned. The transceiver horn antenna was moved mechanically to scan an image from the porcine skin sample by using two stepper motors. The motors were controlled via a Matlab programme and the scanner required 10 min to complete a full scanning cycle from each porcine skin sample. The data (complex scattering parameter (S_11_) or the input reflection coefficient) was obtained directly and saved to be processed using SAR image processing algorithm [46]. The front and the bottom view of the active microwave and MMW scanner are illustrated in Figure 2.

### 2.4. Methodology of Scanning Images

The complex scattering parameter, S_11_, for a flat foam background and a flat metal plate was initially measured without locating any skin sample on the scanner as a part of calibration process. These measurements were performed to cancel the internal reflection effect and to de-convolve the antenna response respectively.

A sample of porcine skin was placed and aligned on the platform of the scanner. Initially, images were scanned from the skin without dressing materials. Then a five-layer gauze burn bandage was placed on the skin surface and images were scanned for the skin with dressing materials. After that, a digital hotplate (type: LED digital hotplate magnetic stirrer, manufacturer: SciQuip Ltd., Shropshire, United Kingdom) was heated to 280 °C and the middle part of the porcine skin sample was placed in contact with the hotplate for 3 min to perform burn. Then the sample with burn was placed and aligned to be scanned. After that, a five-layer gauze burn bandage was placed over the skin with burn and images were scanned for the sample with the presence of dressing materials.

### 2.5. Synthetic Aperture Radar Algorithim

The SAR image processing algorithm developed by Sheen et al. [46] was used in this paper after it has been reduced into two dimensions (range and lateral position). The scattered signal *S* received back to the transmission horn at position located at (x,0) can be considered as the sum of the all scattered signals from each element of the target $f\left(x^{\prime },z^{\prime }\right)dx^{\prime }dz^{\prime }$*,* situated at position $\left(x^{\prime },z^{\prime }\right)$, each with its own phase factor determined by the distance *r* of the element to the horn antenna [42]:

(1) $$S\left(x,k\right)=\int dx^{\prime }\int dz^{\prime }f\left(x^{\prime },z^{\prime }\right)e^{2irk},$$

The wavenumber k is the ratio between the angular frequency ω=2πf, and the speed of the light *c*:

(2) $$k=\frac{\omega }{c},$$

From the dispersion relation for the electromagnetic plane waves; the wavenumber k can be expressed as [46]:

(3) $${\left(2k\right)}^{2}=k_{x}{}^{2}+k_{z}{}^{2}$$

where, $k_{x}$ and $k_{z}$ can be defined using the wavenumber k and the angle θ [42]:

(4) $$k_{x}=2k\sin\left(\theta \right),$$

(5) $$k_{z}=2k\cos\left(\theta \right),$$

The angle θ can be defined by using the distance *r* from the target to the horn antenna as [42]:

(6) $$x^{\prime }-x=r\sin\left(\theta \right),$$

(7) $$z^{\prime }=r\cos\left(\theta \right),$$

Using Equations (4)–(7), the detected signal in Equation (1) can be described as follows [42]:

(8) $$S\left(x,k\right)=\int dx^{\prime }\int dz^{\prime }f\left(x^{\prime },z^{\prime }\right)e^{i\left[k_{x}\left(x^{\prime }-x\right)+k_{z}z^{\prime }\right]},$$

Taking 1-D Fourier transform with respect to $x^{\prime }$ of both sides of Equation (8) gives [42]:

(9) $$T\left(k_{x},k\right)=\int dx\;S\left(x,k\right)e^{ik_{x}x}=\int e^{ik_{z}z^{\prime }}dz^{\prime }\int dx^{\prime }\int dxf\left(x^{\prime },z^{\prime }\right)e^{ik_{x}x}e^{i\left[k_{x}\left(x^{\prime }-x\right)\right]},$$

Due to *x* variation cancelling out, Equation (9) can be simplified as:

(10) $$T\left(k_{x},k\right)=\int e^{ik_{z}z^{\prime }}dz^{\prime }\int e^{ik_{x}x^{\prime }}dx^{\prime }f\left(x^{\prime },z^{\prime }\right),$$

The function $T\left(k_{x},k\right)$ in Equation (10) represents the 2-D Fourier transform of the required target function $f\left(x^{\prime },z^{\prime }\right)$ and therefore it is possible invert this equation using the inverse 2-D Fourier transforms in order to extract the structure of the target as follows [42]:

(11) $$f\left(x^{\prime },z^{\prime }\right)=\int e^{-ik_{z}z^{\prime }}dk_{z}\int e^{-ik_{x}x^{\prime }}dk_{x^{\prime }}T^{\prime }\left(k_{x},k_{z}\right),$$

The function $T\left(k_{x},k\right)$ in Equation (10) is a function of k and it can be converted into a function of $k_{z}$; $T\left(k_{x},k_{z}\right)$ using the following expression:

(12) $$k_{z}=\sqrt{4k^{2}-k_{x}{}^{2}},$$

Similarly, for equally spaced data set Fast Fourier Transform and Inverse Fast Fourier Transform can be applied to extract the target structure function $f\left(x^{\prime },z^{\prime }\right)$.

In the SAR image processing, the reflected radiation are sampled according to the Nyquist criterion [46]. In the frequency domain, the sampling interval is defined by the interval that brings the phase shift between any two successive frequencies to be less than 2π in radian as the obtained data consisted of a real part and an imaginary part. Based on this criterion, the number of samples N for a given bandwidth BW is [46]:

(13) $$N>\frac{2d_{max}}{\left(\frac{c}{2nBW}\right)},$$

where *c* is the speed of the light, *n* is the refractive index of the medium, and $d_{max}$ is the maximum range. Equation (13) indicates that two frequency samples are required per a range resolution, where the range resolution (*R*) of the radar system is defined as [46]:

(14) $$R=\left(\frac{c}{2nBW}\right),$$

### 2.6. Methodology of Data Processing

**Figure 3:**
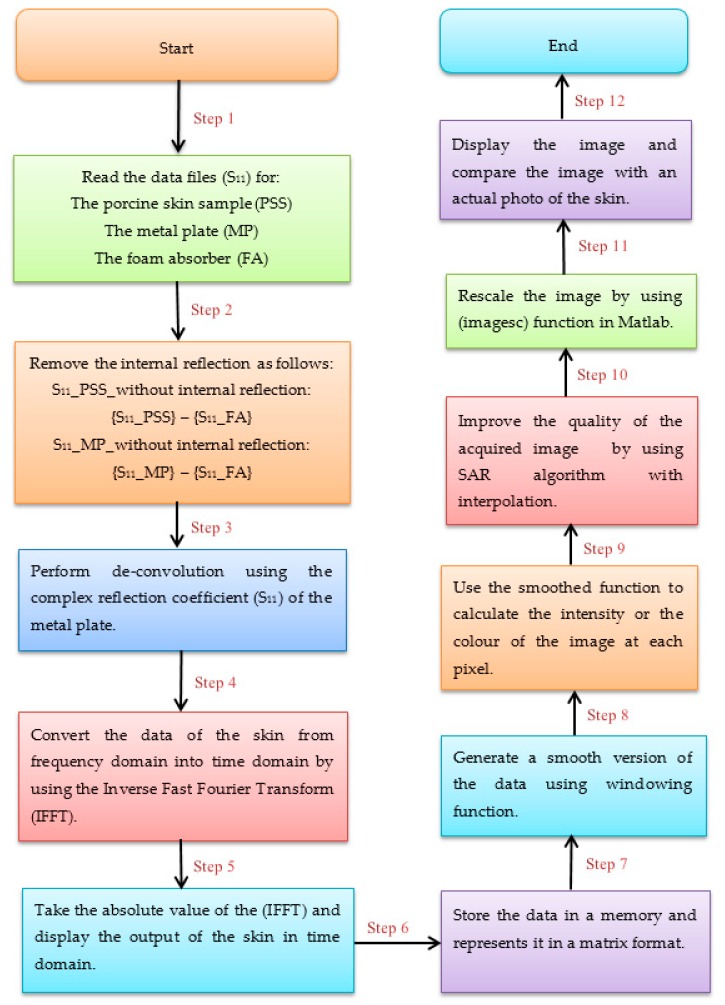
A methodology applied on the data collected from the microwave scanner to obtain an image.

The following block diagram (Figure 3) summarises the methodology of data processing that applied on the measured complex reflection coefficient S_11_:

### 2.7. Methodology of Identifying Artefacts

In radar, a target object (porcine skin) is illuminated by a spatially and temporarily coherent wave source. The reflections from the target are processed through algorithms into an image. The level of reflected radiation from the target is dependent on the target structure and size. The so-called radar clutter is the return reflections from the environment and all objects which are not the target. The radar system is usually signal to clutter limited in the performance [47,48]. The SAR image processing algorithms are used to generate high-quality images. In general, the acquired images consist of desired information, artefacts from signal processing, and unwanted echoes (clutter and speckle). In order to obtain a deeper understanding of these images, it is essential for the user to identify artefacts that manifestations of the technique and that are not present in the target, i.e., (porcine skin). In the experimental work conducted in this paper, the measurements were performed indoors, in an anechoic environment. This minimised the multipath reflections and so helped to reduce the clutter. Furthermore, the acquired images from the scanner were compared with optical photos obtained from the samples, so artefacts can be identified.

## 3. Experimental Results

This section presents images obtained from porcine skin samples over the frequency band 15 GHz to 40 GHz. The images were obtained from samples with and without dressing materials and before and after the application of localised heat treatments. Experimental images were obtained by using the active scanner in Figure 2 and the methodology described in Figure 3.

### 3.1. Images for the Skin without Burns

**Figure 4:**
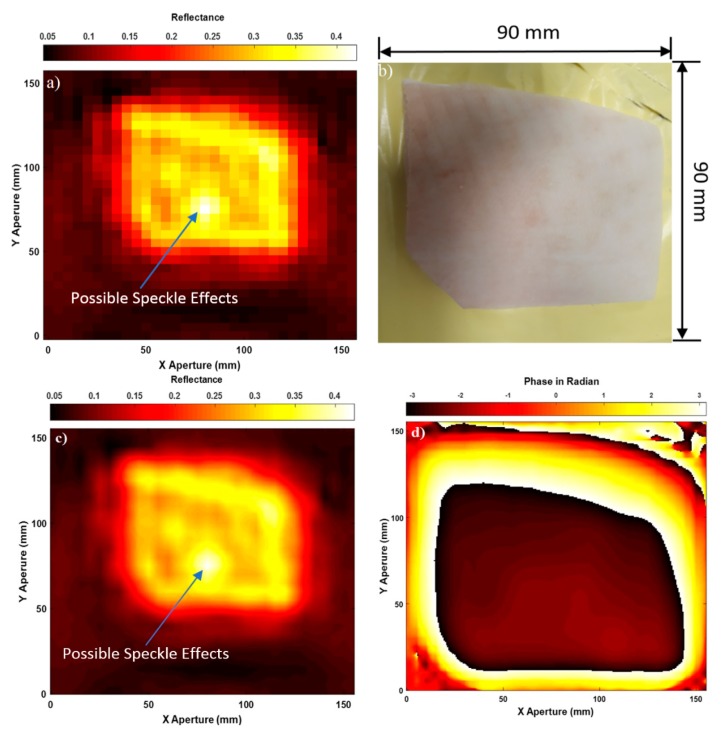
Microwave images for unburned skin over the band 15–40 GHz; case (**a**) represents SAR microwave image 32 × 30 pixels for unburned skin obtained from the amplitude of S_11_, (**b**) represents the sample photo, (**c**) represents SAR microwave image with interpolation 249 × 233 pixels obtained from the amplitude of S_11_, and (**d**) represents SAR microwave image with interpolation 249 × 233 pixels obtained from the phase of S_11_.

Experimental images for porcine skin sample (having length = 90 mm, and width = 90 mm) without burns are illustrated in Figure 4.

Experimental images in Figure 4 indicate that active microwave scanner is capable of scanning images for the skin surface of the porcine skin sample by using both amplitude and phase information of the measured scattering parameter S_11_. The acquired images from the amplitude measurements in (a) and (c) indicate that interpolation enhances the quality of the images significantly, as features on the skin surface can be seen such as edges, bends, and irregularities in the sample shape. The acquired image from the phase measurements in (d) is not dissimilar from that obtained from the amplitude measurements in (c). These results indicate that the two physical quantities (amplitude and phase) are rich in information. 

The unwanted spatial variations of brightness across the images in Figure 4a,c are caused by speckle; this is a result of constructive and destructive interference from the coherent source of radiation and it is most noticeable at limits close to the spatial resolution [49]. Speckle noise is an undesirable effect and it is a system phenomenon, i.e., radar and SAR, and it is not the result of spatial variation of average reflectivity of the radar illuminated surface [50].

### 3.2. Images for the Skin with Dressing Materials

**Figure 5:**
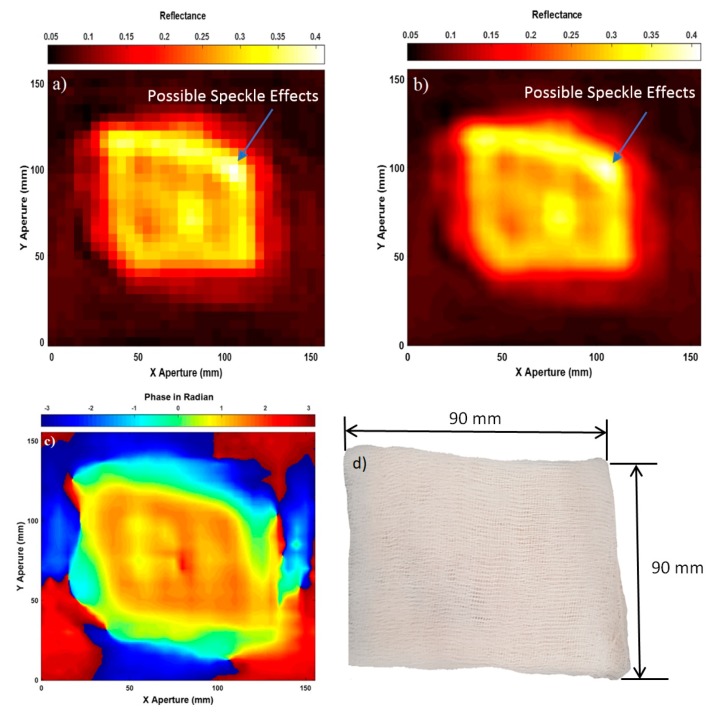
Microwave images for the skin with dressing materials over the band 15–40 GHz; case (**a**) represents SAR microwave image 32 × 30 pixels for the skin with dressings obtained from the amplitude of S_11_, (**b**) represents SAR microwave image with interpolation 249 × 233 pixels obtained from the amplitude of S_11_, (**c**) represents SAR microwave image with interpolation 249 × 233 pixels obtained from the phase of S_11_, and (**d**) represents the skin with dressing materials photo.

Experimental images for porcine skin sample (length = 90 mm, and width = 90 mm) covered with a five-layer gauze burn bandage are illustrated in Figure 5.

Experimental images in Figure 5 indicate that active microwave radiation is capable of penetrating dressing materials as the skin surface is seen through a five-layer gauze burn bandage in images (a), (b), and (c). This can be observed by comparing the obtained images in (a), (b), and (c) with the photo of the sample before adding the dressing materials in Figure 4b. This comparison indicates that dressing materials are highly transparent (transmission in the range of 80–99% depending on the thickness of the bandage as reported in [2,21]) to microwave radiation as the obtained images of the skin with dressing materials show clearly the skin. This indicates that active microwave radiation has a high penetration capability through dressing materials and as a result, this allows the scanner to form high-quality images that show small details in the skin surface of the sample such as edges and irregularities with the presence of dressing materials. The image obtained from the phase measurements in (c) is similar to that obtained from the amplitude measurements in (b).

### 3.3. Images for the Skin with Burns

**Figure 6:**
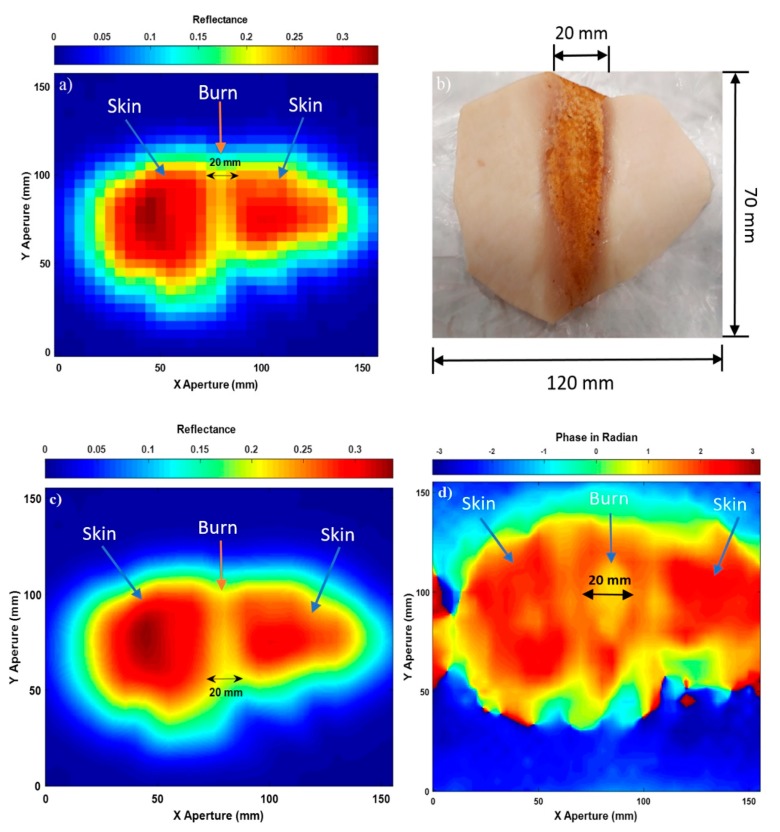
Microwave images for burn-damaged skin over the band 15–40 GHz; case (**a**) represents SAR microwave image 32 × 30 pixels for the skin with burns obtained from the amplitude of S_11_, (**b**) represents the skin with burns photo, (**c**) represents SAR microwave image with interpolation 249 × 233 pixels for the skin with burns obtained from the amplitude of S_11_, and (**d**) represents SAR microwave image with interpolation 249 × 233 pixels for the skin with burns obtained from the phase of S_11_.

Experimental images for porcine skin sample (length = 120 mm, and width = 70 mm) with burns are illustrated in Figure 6.

Experimental images in Figure 6 indicate that the active microwave scanner is capable of distinguishing between the skin without burns (red colour) and the burn-damaged skin (yellow colour in the middle region). The mean reflectance of the skin without burns was found to be 0.28, whereas the mean reflectance of the skin with burns was found to be lower by 0.08. This is due to the burning process that evaporates water from the skin and thereby reducing the reflectance of the burn-damaged skin to 0.2. The scanned images in (a) and (c) show the general shape of the sample and the width of the burn to be 20 mm. This value is similar to the measured width of the burn (20 mm) that was administered by the localised heat treatment.

The image in Figure 6d is obtained from the phase information of the measured input reflection coefficient (S_11_). This image looks different compared with the images obtained from the amplitude measurements in (a) and (c), which is perhaps to be expected, as this is measuring a slightly different quantity.

### 3.4. Images for the Skin with Burns and Dressing Materials

**Figure 7:**
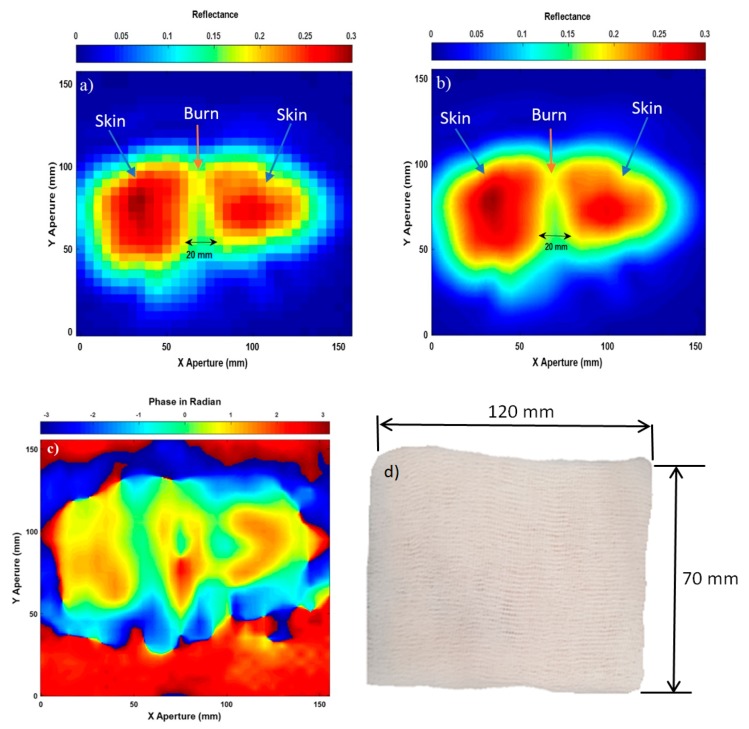
Microwave images for the skin with burns and dressing materials over the band 15–40 GHz; case (**a**) represents SAR microwave image 32 × 30 pixels for the skin with burns and dressings obtained from the amplitude of S_11_, (**b**) represents SAR microwave image with interpolation 249 × 233 pixels obtained from the amplitude of S_11_, (**c**) represents SAR microwave image with interpolation 249 × 233 pixels obtained from the phase of S_11_, and (**d**) represents the skin with burns and dressing materials photo.

Experimental images for porcine skin sample (length = 120 mm, and width = 70 mm) with burns and a five-layer of gauze burn bandage are illustrated in Figure 7.

Experimental results in Figure 7a,b indicate that the images obtained from the amplitude measurements of the input reflection coefficient (S_11_) are capable of detecting burns under a five-layer burn gauze bandage. This is potentially useful as a wound may be monitored without the removal of the protective bandage. The images also provide measurements about the burn width 20 mm and they identify clear boundaries and limits between the normal skin and the burn-damaged skin. Furthermore, the images also indicate that adding dressing materials decreases the reflectance of the skin by 0.02. This can be observed clearly by comparing the reflectance of the images in Figure 7a,b to that in Figure 6a,c. This is due to dressing materials that increase the transmission between the air/skin interface and this increases the emissivity of the skin and as a result decreases the reflectance [2]. 

Experimental image for burn-damaged skin obtained from the phase measurements in Figure 7c is not similar to that obtained from the amplitude measurements in (a) and (b). As amplitude and phase are different physical quantities, it is reasonable to find similarities and differences in the obtained images from those two quantities. The differences in the phase and the amplitude images suggest that there is more information there to be extracted than has already been in this paper, which will be subject of future research.

## 4. Discussion

This paper presents experimental images obtained from porcine skin samples using an active microwave and MMW scanner effective over the band (15–40) GHz. The results obtained from these images in Figure 4, Figure 5, Figure 6 and Figure 7 indicate that it is feasible to detect features of the skin under dressing materials. These features are burns, irregularities in the skin surface, bends, dimensions of burns, and variations in the reflectance of the skin between unburned and burned regions. This is due to the penetration capability of the active microwave and MMW radiation that allows for the assessment of the skin [51]. These measurements suggest that the active microwave and MMW scanner might be an efficient method for monitoring the wound healing progress under dressing materials without necessity of dressings removal.

Experimental images obtained from the phase information of the input reflection coefficient S_11_ in Figure 4, Figure 5, Figure 6 and Figure 7 indicate that the phase values of the images are ranging between −3.14 to +3.14 radian, this is equivalent to ±180 degree and this value is an indication of transferring from less condense medium (air) to an optically denser medium (in this case the skin) [52]. Experimental images obtained from the amplitude and the phase of the input reflection coefficient indicates that the two physical quantities are useful for providing information about the skin and the burns.

Experimental images in Figure 5 and Figure 7 show that skin and burn are detected and observed through dressing materials. The measurements also indicate that there are differences in the mean reflectance values between unburned and burn-damaged skin (0.08). These results indicate that the active microwave imaging system is feasible for distinguishing between skin and burns under dressing materials. Table 1 summarises the mean reflectance values of unburned and burned skin in Figure 4, Figure 5, Figure 6 and Figure 7.

In our previous research [21], we have investigated the feasibility of using active millimeter-wave radar for measuring the optical path length of different types of dressing materials coated with medicinal cream and water. Experimental measurements in [21] indicate that active millimeter-wave radar can provide precise information about the optical path length of the dressing materials and hand support cast. The results obtained from this study [21] has led us to use the active microwave scanner and SAR image processing algorithm in this paper to scan images from porcine skin samples with the presence of dressing materials. These images aim to demonstrate the capability of active microwave and millimeter-wave technology to be used for burn wounds diagnostics under dressing materials. The results obtained from the active scanner in this paper, indicate that SAR images are capable to distinguish between unburned and burned skin under dressing materials as well as features of the skin such as burn width, irregularities and edges on the skin surface.

Due to the grown interest of passive sensing technology, we have investigated the signature of the human skin over the millimeter wave band 80–100 GHz using non-contact sensor (radiometry) [22,23,36,37,38]. Radiometric measurements performed on human skin [22,23,36,37,38] indicate differences in the mean emissivity values of the skin between individuals, locations on the body, genders, and normal and wet skin. These studies [22,23,36,37,38] reveal that these variations are related to the skin thickness and water content, a capability that enables radiometry to be used as a non-contact sensor to detect and monitor skin conditions such as eczema, psoriasis, malignancy, and burn wounds. Further, radiometric measurements performed on animals tissues (phantom chicken and porcine skin) in [2,27] indicate that radiometric sensitivity is sufficient to distinguish between unburned and burned skin in tens of seconds. These measurements [2,22,23,27,36,37,38] and the images presented in this paper indicate that active and passive microwave and millimeter-wave technologies are feasible to be used for monitoring the wound healing under dressing materials.

The system depicted in Figure 2, i.e., the scanner, represents a technology readiness level (TRL) three system; a proof of concept scanner for initial capability demonstrations. To progress the technology to the higher TRLs where measurements could be made on patients would require appropriate redesign of the active scanner such that arrays of antennas can be used and the measurements can be performed in tens of seconds. Measurements with these follow-on demonstrators would be made with greater precision and convenience, offering a rapid and non-invasive diagnostic technique in critical situations where the removal of dressing materials might potentially cause damage to the neo-epithelium covering the wound bed and increase the risk of infection.

The next progress in this area of researches needs expansion of experimental investigations onto real burn damages of alive human skin. The mean reflectance values from people with different burns injuries are needed to be measured and identified and then compared with the mean reflectance values of healthy skin. Any deviations from the standard norms should be identified as well as unusually high or low levels of reflectance values. The lower reflectance values of burn-damaged skin are indicative of a dry burn, whereas the higher reflectance values are indicative of the presence of exudates, infection or a non-healing state of the skin. When these measurements are performed, a machine learning algorithm can be developed to classify these images without the need of optical photos.

In this paper, we are aiming to provide a proof-of-concept and to demonstrate a capability of the active microwave scanner to be used for monitoring the wound healing under dressing materials without necessity of dressing removal. As a plan for future work, a recommendation is made to acquire images from patients having different degrees of burn injury. Images should be obtained with and without the presence of dressing materials to allow comparisons. Then the images can be compared with other images taken at different days (change detection). This might be useful for identifying signs of infection (exudates and swelling) as well as detecting changes on the skin surfaces during the wound healing process without the removal of the dressing materials.

## 5. Conclusions

A synthetic aperture radar imaging system with 25 GHz bandwidth and 6 mm spatial resolution was used to scan images from porcine skin samples. The system was calibrated using known targets with known reflection properties and these were: (1) a flat metal plate, and (2) a flat microwave foam absorber. The calibration measurements of the metal plate were used for de-convolution and dispersion compensation, whereas the calibration measurements of the foam absorbing materials were used for internal reflection removal.

Active microwave and MMW images acquired over the band 15–40 GHz from porcine skin samples with the presence of dressing materials indicate that skin and burns are observed through a five-layer gauze burn bandage. Experimental images obtained from porcine skin samples indicate that there is useful information in the amplitude and phase measurements of the input reflection coefficient (S_11_). These images demonstrate the potential of using SAR images for burn wounds diagnostics under dressing materials.

## Figures and Tables

**Table 1:** Summary of the mean reflectance values of the unburned and burned skin.

Sample Description	Mean Reflectance of the Skin	Mean Reflectance of the Burn
Sample 1: Skin without Dressing Materials; Figure 4	0.32	-
Sample 1: Skin with Dressing Materials; Figure 5	0.30	-
Sample 2: Skin with Burns and without Dressing Materials; Figure 6	0.28	0.20
Sample 2: Skin with Burns and Dressing Materials; Figure 7	0.26	0.18
